# Parallel-Distinct Structures of Internal World and External Reality: Disavowing and Re-Claiming the Self-Identity in the Aftermath of Trauma-Generated Dissociation

**DOI:** 10.3389/fpsyg.2017.00216

**Published:** 2017-02-17

**Authors:** Vedat Şar

**Affiliations:** Department of Psychiatry, Koc University School of Medicine (KUSOM)Istanbul, Turkey

**Keywords:** childhood abuse, childhood neglect, borderline personality, dissociation, somatic symptoms, psychological dissociation, identity integration, psychotherapy

## Abstract

The nature of consciousness and the autonomy of the individual's mind have been a focus of interest throughout the past century and inspired many theories and models. Revival of studies on psychological trauma and dissociation, which remained outside mainstream psychiatry, psychology, and psychoanalysis for the most part of the past century, has provided a new opportunity to revisit this intellectual and scientific endeavor. This paper attempts to integrate a series of empirical and theoretical studies on psychological consequences of developmental traumatization, which may yield further insight into factors which threaten the integrity of human consciousness. The paper proposes that an individual's experience of distorted reality and betrayal precipitates a cyclical dynamic between the individual and the external world by disrupting the developmental function of mutuality which is essential for maintenance of the integrity of the internal world while this inner world is in turn regulated vis-à-vis external reality. Dissociation -the common factor in all types of post-traumatic syndromes- is facilitated by violation of boundaries by relational omission and intrusion as represented by distinct effects and consequences of childhood neglect and abuse. Recent research conducted on clinical and non-clinical populations shows both bimodal (undermodulation and overmodulation) and bipolar (intrusion and avoidance) neurobiological and phenomenological characteristics of post-traumatic response. These seem to reflect “parallel-distinct structures” that control separate networks covering sensori-motor and cognitive-emotional systems. This understanding provides a conceptual framework to assist explanation of diverse post-traumatic mental trajectories which culminate in a common final pathway comprised of partly overlapping clinical syndromes such as complex PTSD, dissociative depression, dissociative identity disorder (DID), or “borderline” phenomena. Of crucial theoretical and clinical importance is that these maladaptive post-traumatic psychological formations are regarded as processes in their own right rather than as a personality disorder innate to the individual. Such mental division may perform in that internal detachment can serve to preserve the genuine aspects of the subject until such time as they can be reclaimed via psychotherapy. The paper attempts to integrate these ideas with reference to the previously proposed theory of the “Functional Dissociation of Self” (Şar and Öztürk, 2007).

As a relational being, individual and his/her psychological survival is very much affected by the social environment which may be less than optimal for adjustment. Hence, the integrity of human consciousness and the autonomy of the individual's mind are densely interwoven with psychological trauma and its consequences. Psychological trauma refers to psychological injury, wound, and pain. The present paper proposes that analogous to the physical response to overwhelming threat to homeostasis, the psychological response to traumatic stress comprises *protection* of vital parts of the psychobiological system, even at the cost of other parts, until the threat is over. Thus psychosocial adjustment, in developmental periods of life in particular, may constitute arrest or delay in realization of one's unique and authentic potentials which necessarily leads to a *division of mind*. The consequence is disavowal of or inability to develop one's *integrated self-identity* with the possibility to subsequently re-claim it under favorable conditions.

Healing of the psychologically wounded individual is based on the *congruent sequence of discourse, theory, model, technique, and application*, where none of these can replace any of the others. Hence this paper begins with theoretical assumptions before addressing empirical material in the later sections. Several hypotheses are revived in the context of chronic developmental trauma, the very trajectory which constitutes a threat to psychological survival of the individual.

## Trauma and “split-mind”: functional dissociation of self

In an inquiry into the interface between individual and society, Şar and Öztürk (2007, 2013) proposed the concept of the personal “Sociological Self” as part of a theory which addresses both non-clinical and post-traumatic conditions. According to their theory of “Functional Dissociation of Self,” expansion of the individual's sociological self due to developmental traumatization (e.g., childhood neglect or abuse) and additional factors (e.g., overadjustment to social demands of benign or malevolent types) leads to a detachment in the individual's internal world. This theory claims that this division has a protective aim; i.e., individual's “sociological self” is devoted to save his/her “psychological self” from annihilation (where the latter represents the unique and authentic aspects of the individual).

In his famous study “Sickness Unto Death” (published in 1849), Danish philosopher Kierkegaard (1849/1983) assumed that the human existence was based on relation (although not necessarily an interpersonal one): “A human being is a spirit. But what is spirit? Spirit is the self. But what is the self? The self is a relation that relates itself to itself or is the relation relating itself to itself in the relation” (Marino, 2009). To augment Kierkegaard's statement, one could better say that, an “integrated self” is nothing else than the harmonical relation between the “sociological self” and “psychological self” of the individual as proposed and conceptualized by Şar and Öztürk (2007, 2013). After detaching from sociological self, the “psychological self,” however, becomes frozen and lacks the resources for further development. In addition to the detached “sociological” and “psychological” selves, the “trauma-self” Şar and Öztürk (2005, 2007) emerges and remains in a perpetual status of help-seeking. In fact, this is the “symptomatic” self (Öztürk and Şar, 2016a) which may be explicit in daily life.

Self and/or identity disturbance, emptiness, and fear of abandonment seem to reflect such conditions (Meares et al., 2011). The central state is “painful incoherence” as observed in a “dissociative depression” (Şar, 2011a). Alternatively, while describing loss of connection to the “psychological self,” the Turcic poet Yassawi (1093–1166) stated: “I can not get news from myself.” Similar testimonies also feature in the text *Feeling Unreal: Depersonalization Disorder and the Loss of the Self* (Simeon and Abugel, 2006).

In fact, the idea of “split mind” and potential co-existence of healthy and pathological parts is not new as Bleuler's (1911) concepts of “schizophrenia” and “double book-keeping” and Winnicott (1965) conception of the “false self” reveal. The Theory of Functional Dissociation of Self (Şar and Öztürk, 2007) is contrasting however in not regarding such division as necessarily pathological or maladaptive; i.e., a harmonical relationship between sociological and psychological selves is possible and, even, healthy. Hence such dual development may also have normative (“non-traumatic”) aspects. Description of the optimal trajectories of both selves throughout each phase of life remains an important task which exceeds the aims and limits of the present paper.

## Complex trauma and psychiatric consequences: personality or disorder?

Cumulative relational traumatization in the early years of life has been differentiated from acute stressful events as “complex trauma” (Herman, 1992; Van der Kolk, 1996; Courtois, 2004). Research shows that chronic relational traumas (e.g., childhood abuse and neglect) are as damaging to mental health and relationship outcomes as the non-relational and acute ones (Anders et al., 2012). Various types of complex clinical constellations may emerge in the aftermath of relational trauma such as complex posttraumatic stress disorder (PTSD), dissociative depression, dissociative identity disorder (DID), and “borderline” phenomena (Şar, 2011b, 2014, 2016).

Due to their pervasive and enduring character, these syndromes were for most of the twentieth century considered by mainstream psychiatry, psychology, and psychoanalysis as disturbances of personality. Unlike earlier theories which assumed a strong relationship between personality and maladaptation (Kernberg, 1987), I propose a clear demarcation between unique aspects of the individual and trauma-related psychological process which “intrudes” and/or “possesses” the subject's “internal world.” Nor is this merely a point of scientific curiosity; I further contend that *development of a clear vision about this demarcation line by the traumatized individual is necessary in treatment*. The associated “damage” is principally reversible in conditions where “repair” (i.e., appropriate treatment) is available.

To consider this theoretical stance, and to inquire into various aspects of such trauma-generated mental division of this kind, the paper reviews findings from a limited series of hypothesis-generating empirical studies which gathered data on lifelong consequences of childhood trauma in various clinical and non-clinical populations. I hypothesize that chronic developmental traumatization leads to a functional re-organization of the mind into enduring “parallel-distinct structures” which operate side by side without being fully integrated with each other in aim, content, and process. Such re-organization serves in buffering the traumatic breach in and between external reality and internal world. Although devoted to protection of the individual's unique psychological aspects (i.e., rather than constituting a mere fragmentation in the face of relational trauma), such division of the internal world may undermine the enduring experience of a coherent self-identity. It may also lead to crisis-proneness due to loss of mutuality between “parallel-distinct structures.”

## Stimulus modulation and trauma: broken boundaries and loss of integration

Due to his professional background in physiology, Freud (1920/1975) assumed that the central nervous system was protected by a “shield” (“Reizschutz”) in the sense of a stimulus barrier defending from excessive stimulation. In traumatic conditions, the barrier is suddenly and violently breached, overwhelming the nervous system with an influx of stimuli it cannot handle. *Recent neurobiological research has documented the fact that traumatic stimuli trigger two types of reaction* (Lanius et al., 2010). One is primarily *avoidant*, and constitutes emotional overmodulation based on excessive corticolimbic inhibition. This can lead to subjective disengagement from content of the traumatic memory. The other is predominantly *intrusive*, and a hyperaroused type of response which comprises undermodulation of emotions which may lead to re-experiencing the traumatic event in the “internal world.”

Stimulus modulation is an idea with diverse repercussions. From a physiological perspective, chronic overstimulation may lead to habituation or exhaustion. In contrast, denervation and other forms of stimulus deprivation cause an increase in the magnitude of subsequent responses (a phenomenon commonly referred to as denervation supersensitivity).

From a psychological perspective, stimulus deprivation may weaken the individual's orientation to reality because the person is not buffered against the predominance of “internal stimuli.” Disproportionate stimulation interferes with the mental capacity to develop links between thoughts, emotions, and behaviors; i.e., *it provokes dissociation* (Şar and Öztürk, 2013). Hence, stimulus modulation is essential for maintaining homeostasis and healthy adaptation; i.e., keeping the “integrative mode of consciousness” alive (Winkelman, 2011).

## Psychological survival by mental avoidance: the vicious cycle

Much of the trauma-related dysfunctions are “attempted solutions to dilemmas which are more focused on survival than recovery” (Briere, 2002). For instance, when confronted with an imminent life-threat for which flight-or-fight is no longer an option to counter danger, the organism may shift to immobility. To escape the threatening situation as well as the internal distress and arousal, the response of “shutdown dissociation” may be adaptive for survival (Schalinski et al., 2015) leading to freezing or submission.

Many traumatized patients continuously wish to “forget” their disturbing experiences without being able to do so. In fact, *intrusion* and *avoidance* constitute a vicious cycle rather than totally independent phenomena. Representing a “rebound” phenomenon, avoidance or suppression can counterproductively lead to the state of mind one had hoped to avoid (Wenzlaff and Wegner, 2000); i.e., an increased tendency to experience mental intrusions as well as diminished behavioral control (Abramowitz, 2001). Suppressing thoughts can lead to an increase in behavior related to the formerly suppressed thought (Erskine, 2008; Erskine and Georgiou, 2008). Thought suppression has been implicated as an etiological and/or maintaining factor in depression, generalized anxiety disorder, specific phobia, obsessive-compulsive disorder, and PTSD (Purdon, 1999). In a general population study, suicidality, substance abuse, dissociation, and problematic activities such as self-injury and dysfunctional sexual behaviors were all indicators of a robust latent variable; namely, dysfunctional avoidance (Briere et al., 2010).

Avoidance and intrusions of traumatic mental content constitute the basic clinical components of PTSD. These usually co-occur on an individual basis as do the two types of response. As the bipolar character of the constellation represents a detaching of mental contents or even somewhat enduring “structures” which are “phobic” to each other (Van der Hart et al., 2006), some authors claim that PTSD is, in fact, a dissociative disorder (Nijenhuis, 2014). Alternatively, a dissociative subtype of PTSD has been proposed (Lanius et al., 2010) to delineate those post-traumatic conditions which are characterized by overmodulation of emotions. The latter conceptualization has been adopted in the DSM-5 (American Psychiatric Association, 2013) on the basis of depersonalization and/or derealization experiences as accompanying symptoms of PTSD. Debate on the accuracy of these two stances is still ongoing. This is, because, like PTSD, dissociative disorders are characterized not only by avoidance (“negative” dissociative symptoms) but also by intrusions (“positive” dissociative symptoms).

Alternatively, identity alterations observed in dissociative disorders may be considered an elaborated version of trauma-related mental *intrusions* and *avoidance* which correspond to the basic mechanism of PTSD. Unlike in PTSD, however, in DID and its subtreshold forms, traumatic memories are widely “decontextualized” (Brewin, 2001) and processed to retain internal and external balance (Öztürk and Şar, 2016b). Further, this leads to formation of “discrete behavioral states” (Putnam, 1997) each with their own sense of self and agency, personal history, and mission, subsequent to an elaboration based on trauma-related cognitions, compensatory structures, and emotions assigned to them. Namely, DID and PTSD may precede each other chrononologically or may be co-present depending on the status of the patient in the spectrum between avoidance or intrusion.

Anxiety-laden PTSD can also account for treatment-seeking in a state of crisis in context of DID. For example, in a case of “vampirism” with coexisting PTSD and DID, the “host personality” was suffering from flashbacks due to PTSD while a disillusioned-traumatized “child” alter personality triggered violent attacks to be carried out by an aggressive part (Sakarya et al., 2012). Being unaware of the inner cycle, the patient was suffering from temporary loss of control following increased “appetite” for blood. Diminished dissociative barriers may also lead to emergence of PTSD symptoms during treatment of DID which were lacking initially.

*Dissociation is a constant feature of post-traumatic conditions independent of the main psychiatric diagnosis* (Şar and Ross, 2006; Şar, 2011b). For example, peritraumatic dissociation is known as a predictor of PTSD (Van der Hart et al., 2008). While acute trauma can induce peritraumatic dissociation, complex trauma leads to chronic dissociation more readily (Ogawa et al., 1997). In a study on the general population, while childhood interpersonal (complex) trauma was related to altered self-capacities (interpersonal conflicts, idealization-disillusionment, abandonment concerns, identity impairment, susceptibility of influence, affect disregulation, and tension reduction behaviors), non-interpersonal traumas and adult traumas were typically unrelated to the scales assessing these features (Briere and Rickards, 2007). The main reason for this different effect is *adaptation to trauma in the developmental years*—a response of the whole organism—which has vital importance for the survival of the growing individual.

In tandem with the notion of a protective detachment of mind proposed in the present paper, Schimmenti and Caretti (2016) state that “*…dissociation may paradoxically protect the traumatized child from a fragmentation of the self through multiple disconnections in the self, occurring at both mental and bodily levels.”* In fact, such paradox can be explained only by a duality-based model of mind allowing detachment in a level other than alternate personality states: i.e., *it is the detached “sociological” self which is fragmented* Şar and Öztürk (2005, 2007). Bromberg (1998, 2011) has proposed that dissociation could be considered as a *proactive strategy* used by children to protect themselves from developmentally traumatic interactions with caregivers that disrupt the sense of continuity in the self regardless of its natural multiplicity.

A recent MRI study (Mutluer et al., in press) conducted on adolescent girls with PTSD due to severe sexual abuse yielded bilateral decrease in volumes of amygdala, anterior cingulate, hippocampus, and diminished thickness of prefrontal cortex compared to the healthy controls. However, analyses within the PTSD group revealed a tendency of *lateralization* in findings showing significant negative correlations between clinical symptoms of Simple and Complex PTSD and volume changes of the right hemisphere subcortical structures. A significant positive correlation was shown between core dissociative phenomena (depersonalization, derealization, and identity alteration which fit the dissociative subtype of PTSD or DID) and *left frontal cortical thickness*. These findings led the authors to support the idea that dissociation may be neuroprotective (Ross et al., 2015) at least until adolescence. In the healthy group, the structures of right and left hemispheres were better correlated with each other. In support of the observations previously made by Farina et al. (2014), this symmetrical appearance of correlations suggested better *connectivity* of the brain in the healthy group. Unlike dissociation, denial or minimization of childhood trauma seemed to be “toxic” in that it was negatively correlated with the thickness of the right prefrontal cortex (Mutluer et al., in press).

## Dissociation to “parallel-distinct structures” and narrowed consciousness

The cardinal feature of dissociation is disruption of usually integrated mental functions of consciousness, memory, identity, emotion, perception, behavior, cognition, and/or motor activities (American Psychiatric Association, 2013). Such disruption may lead to discontinuities in sense of self and agency which cause a disturbance of self-identity. In his 1889 dissertation, “L'automatisme psychologique,” Pierre Janet stated that automatisms were the most elementary form of psychological functioning (Van der Hart and Horst, 1989). Adaptive operations of the evolutionarily bestowed simple and complex psycho-biological action systems (e.g., attachment, sexuality, care-taking, socialization etc.) constitute the functioning of both normal and dissociative individuals (Van der Hart et al., 2006). From a contrasting point of view, post-traumatic dissociation may disrupt both sensori-motor, and cognitive-emotional functions. The so-called BASK (behavior-affect-sensorium-knowledge) model of dissociation underlines this separation of mental functions from each other (Braun, 1988).

I propose that dissociative disruption leads to emergence of “parallel-distinct” mental structures rather than to simple disaggregation. In contrast to the BASK model, such parallel-distinction may have a vertical or horizontal character as valid for the organization of the nervous and mental system in general (Echarte, 2016). The horizontal dissociation points to the altered relationship between higher and lower order brain structures, changing the status of the hiearchical control. Vertical dissociation, however, points to the networks covering all levels of complexity in hierarchy; i.e., from automatisms including sensori-motor responses to the upper levels of consciousness such as sense of self (Meares, 1999; Bob and Faber, 2006).

As well as compartmentalization, dissociation may also lead to detachment. Examples of symptoms of detachment are: emotional numbing, depersonalization-derealization, out-of-body experiences, and dissociative amnesia due to encoding deficit. Those related to compartmentalization are functional neurological (conversion) symptoms, hypnotic phenomena, “made” actions (Schneiderian experiences), multiple identities, and dissociative amnesia due to retrieval deficit. Identity confusion, on the other hand, is a symptom that can be associated with either detachment or compartmentalization (Brown, 2006). Schimmenti and Caretti (2016) have stated that “*compartmentalization and detachment are two sides of the same coin; that is, the psychobiological dysregulation deriving from early relational trauma and the development of competitive self-states that cannot be integrated into consciousness.”*

Recognition of detachment and compartmentalization as distinct types of dissociation has implications for treatment as well. Namely, recent evidence suggests that cognitive behavior therapy, which utilizes an adapted anxiety-disorder model, is an effective treatment for pathological detachment (Hunter et al., 2005). Other forms of treatment may be more appropriate for pathological compartmentalization (Holmes et al., 2005).

Narrowing or alteration of consciousness may also be considered as types of dissociation. In order to differentiate pathological dissociative experiences of “trauma-related altered states of consciousness” (TRASC) from what they considered to be nondissociative forms of psychological distress (that is, symptoms that can be readily experienced within “normal waking consciousness”; NWC), Frewen and Lanius (2014) operationalized a four-dimensional symptomatology called the “4-D model.” This model highlights four dimensions of consciousness affected by traumatic stress, whether in TRASC or NWC-distress form. These are *time-memory* (e.g., flashbacks vs. reminder distress without reliving), *thought* (e.g., voice hearing vs. negative self-referential thinking), *body* (e.g., disembodied experiences of depersonalization vs. embodied experiences of distress), and *emotion* (numbing, affective shutdown vs. non-dissociative forms of negative emotionality such as fear, anxiety etc.). While markers of TRASC and NWC predicted social and occupational impairment in a follow-up study on the acute aftermath of traumatic events, contrary to hypothesis, childhood trauma was more strongly correlated with the symptoms of NWC-distress than with TRASC (Frewen et al., 2015).

The latter finding can be explained by the observation that long-term consequences of chronic developmental traumatization such as Complex PTSD may be represented predominantly by depression and subtle disturbances of self perceived as passive-influence experiences (Şar, 2011a). Constituting resistances of the “trauma-self” (Öztürk and Şar, 2016a), such relatively subtle and chronic phenomena are associated with NWS (i.e., that the individual appears “normal” but not crisis-prone in daily life) rather than TRASC which is related to more explicite dissociation. This condition reflects the secondary phase (“Trauma-Illness” as opposed to “Inflammation” and “Estrangement” phases) of post-traumatic process in the proposed “Tri-Modal (T-MR) Reaction Model of Protection” (Mutluer et al., in press) as inspired by findings of a recent neurobiological study on adolescent girls with PTSD due to severe sexual abuse.

## Childhood neglect and abuse: distinct tracks of post-traumatic response

Childhood traumata can arguably be classified into two types is proposed: those of interpersonal *omission* (neglect) and *intrusion* (abuse). This suggestion is consistent with contemporary understanding of the bimodal nature of neurobiological response to traumatic stress, where overmodulation and undermodulation of emotions are operative (Lanius et al., 2010). Both dichotomies correspond to the bipolar clinical character of all trauma-related syndromes including PTSD: namely, intrusion and avoidance of the traumatic mental content. Treatment needs to take place in the context of a “therapeutic window” between overwhelming exposure and excessive avoidance (Briere, 1996, 2002; Porges, 2011).

Four issues seem to prevent researchers from identifying the distinct impacts of omission and intrusion. First, the naturalistic co-presence of these patterns for a majority of studied populations deflects attention from their distinct cognitive-emotional and sensori-motor consequences. Second, the discrepancies in results of self-report and clinician-administered assessment raise questions about the reliability of these tools. In fact, such discrepancies may represent the “real” condition; namely that both the subjective experience and the objective mental status of the evaluated individual may differ between personal and interpersonal settings. The latter possibility would be consistent with the very nature of dissociation as an interpersonal experience sensitive to betrayal, attachment, and perception. Third, different tracks of post-traumatic processing may be pursued by sensori-motor and, alternatively, by cognitive-emotional dissociation. Fourth, timing of childhood maltreatment (Schalinski and Teicher, 2015) may account for considerable variance in psychological consequences. Therefore, distinct post-traumatic phenomena should be assessed and handled separately even if they occur concurrently.

The partial phenomenological overlap between borderline personality disorder (BPD) and dissociative disorders -which both have a trauma-related origin- has been well-documented by empirical research (Korzekwa et al., 2009; Brand et al., 2016). For example, in a controlled study of a high-functioning college population (Şar et al., 2006), a significant overlap between the two syndromes was substantiated: 72.5% of those with BPD had a dissociative disorder in contrast to only 15.0% of the non-BPD controls. Although some authors consider BPD a dissociative disorder (Meares, 2012), and others take an opposite stance by regarding dissociative disorders in the realm of BPD (Lauer et al., 1993), such overlap does not necessarily discard the possibility of existence of two qualitatively distinct entities.

The question arises whether such overlap is also related to co-existence of different types of developmental trauma. To test this hypothesis, a multivariate statistical analysis allowed Şar et al. (2006) to calculate the distinct relationships between the two syndromes and reports of various types of childhood trauma separately. According to this study, BPD was predicted by overall severity of childhood trauma, childhood sexual abuse (SA), emotional abuse (EA), physical abuse (PA), and physical neglect (PN). Dissociative disorders, on the other hand, were predicted by childhood emotional neglect (EN) and denial (minimization of trauma including idealization of parents) of childhood adversities. Apparently, BPD was related to the *intrusion* (both bodily and psychological) and *bodily omission* whereas dissociative disorders were related to the *psychological omission* only. The two clinical response patterns did not markedly interact according to any childhood trauma measure. Although both syndromes have their own important histories of conceptualization, *the existing empirical data do not rule out the possibility that they represent two types of post-traumatic response*.

Apart from a potential personality disorder, a similar pattern can be observed at the more severe pole of the psychopathological spectrum. In a study on patients with schizophrenic disorder (Şar et al., 2010b), those patients who had high scores of dissociation (and who had elevated negative symptoms, childhood trauma total, and childhood EN scores) were divided into two further subgroups in cluster analysis. The first was characterized by childhood EA (psychological trauma of intrusive type) and predominantly had symptoms associated with DID and positive symptoms. This group, who reported dissociation *directly*, had earlier age of onset and longer duration of the disorder. The second group was characterized by childhood PA and SA as well as by PN (“bodily trauma” of both intrusion and omission types). The latter group had more BPD criteria, somatic complaints, and general psychiatric comorbidity which represented dissociation indirectly.

A recent follow-up study on patients with bipolar disorder (Çakir et al., 2016) documented that among patients who received anticonvulsant treatment, those with elevated childhood EA and PA scores (intrusion) had poorer outcome in terms of relapse (a trait measure). Among omission types of trauma, while PN was related to mean severity of the mood disorder episodes and psychotic features, EN was related to suicide attempts. Hence, unlike the unipolar (dissociative) depression described below, omission type of trauma predicted the severity of the state. The childhood trauma total score was related to psychotic features, the number of lifetime comorbid psychiatric disorders and the number of courses of antidepressant drug treatment. The latter was also associated with comorbid diagnosis of lifetime diagnosis of PTSD. Unlike for the anticonvulsant treatment group, lithium non-responders were more common among patients with lifetime diagnosis of PTSD.

Apparently, *nosological fragmentation* (increased general psychiatric comorbidity) is one of the outcomes of developmental traumatization which is facilitated by total severity of childhood abuse and neglect in bipolar disorder and bodily type of childhood adversity inschizophrenia. This phenomenon requires further addressing via epigenetical research. While reported observations on patients with schizophrenia replicated findings on the relationship between EN (omission) and dissociation in non-clinical populations (Ogawa et al., 1997; Şar et al., 2006), different types of intrusive traumatization (for instance, emotional vs. bodily abuse) seem to have distinct clinical consequences. While bodily intrusion may lead more readily to indirect symptoms of dissociation, emotional intrusion, however, seems to be related to positive symptoms (e.g., hallucinations of voice-hearing type) which constitute the *third person perspective* of consciousness according to the 4-D model (Frewen and Lanius, 2014).

## Embodied trauma and bodily dissociation: clinical implications

Şar (2011a, 2015b) has proposed the term “dissociative depression” for a complex post-traumatic condition which may involve features of BPD and transient feelings of loss of control in addition to the symptoms of unipolar depression and dissociation. In a study among women in the general population, dissociative depression (current major depression comorbid with lifetime diagnosis of dissociative disorder) was predicted by educational deprivation and childhood SA (Şar et al., 2013a). In the same sample, childhood PA, lifetime diagnosis of major depression and/or dissociative disorder predicted functional neurological (conversion) symptoms (a type of sensori-motor dissociation; Şar et al., 2009). While dissociative depression was related to a combination of omission and bodily intrusion of sexual type, PA (bodily intrusion of non-sexual type) added sensori-motor dissociation to this condition possibly due to unassimilated sensori-motor reactions; i.e., somatic memory).

A different appearance was obtained in a study (Kiliç et al., 2014) of women with a physical illness which explored the relationship of post-traumatic anger and cognitive-emotional and sensori-motor dissociation to unipolar depression. The diagnosis of lifetime depressive disorder (a trait measure) was predicted by sensori-motor dissociation whereas cognitive-emotional dissociation was related to current severity of depression (a state measure). The former track was associated with childhood neglect while the latter was related to abuse. While similarly leading to depression as the final common pathway, the consequences of childhood neglect (omission) and abuse (intrusion) were associated with distinct trajectories.

A study on women with back pain or headache (Yücel et al., 2002) found no relationship between childhood adversity and cognitive-emotional dissociation. However, sensori-motor dissociation was predicted by EN; hence the statements “the body keeps the score” (Van der Kolk, 1994) and “embodied” dissociation (Lanius et al., 2014) are valid. The question arises whether the relationship between childhood adversity and its consequences follow a different track among patients with a somatic illness. However, the special relationship between childhood neglect and body perception was reported in a non-clinical college population as well: Both childhood PN and EN were associated with symptoms of bodily depersonalization while childhood abuse did not (Şar et al., 2017b).

A recent case report describing “unperceived (total denial of) pregnancy” (Şar et al., 2017c) ends with the words of the affected young woman who underlines the connection between loss of trust and the feeling of being abandoned by one's own body:

“It is really hard for me to describe how I feel about the denial of pregnancy. I know that, in the weeks following the birth, I asked myself a lot of questions about it. I felt betrayed by my body. I felt like my body was a part of me that I can't control. I thought that if my body did this to me, and hid all these things from me, I could not be certain about anything that I felt, or anything that I thought about me. The main feeling that I had, was to not trust myself anymore.”

Kierkegaard, while inquiring mind-body relationships, was inspiring about the potential protective nature of mental division and dissociative depression. He described “unhappiness” (alias depression) as “*a suffering which must have its basis in a mis-relation between…mind and body, for it has no relation to (one's) spirit, which on the contrary, because of the tension between…mind and body…gain(s) an uncommon resiliency*” (Marino, 2009, para. 9). Vice versa, in his well-known presentation (“Television”), French psychiatrist Lacan (1974/1990, p. 6) pointed to the associative mission of the body in conditions of a detached internal world:

“In fact the subject of the unconscious is only in touch with the soul via the body, by introducing thought into it: here contradicting Aristotle. Man does not think with his soul, as the Philosopher imagined. He thinks as a consequence of the fact that a structure, that of language—the word implies it—a structure carves up his body, a structure that has nothing to do with anatomy. Witness the hysteric. This shearing happens to the soul through the obsessional symptom: a thought that burdens the soul that it doesn't know what to do with. Thought is in disharmony with the soul.”

Nevertheless, one has to be careful in interpreting Lacan's usage of the word unconscious which he anchores in external reality: “*There is no unconscious except for the speaking being,” …“the others, who possess being only through being named—even though they impose themselves from within the real-,”…“it speaks, does the unconscious, so that it depends on language”* (ibid, p. 5). The research on embodied trauma and bodily dissociation, however, has the potential to illuminate the lost link of communication between “unformulated experience” (Stern, 1997) and the illness as expression of it: the *psychosomatics*.

## Being “unable to speak”: in search for a (common) language

Indeed, trauma explicitely disrupts the capacity to develop consistent narratives of experiences (Van der Hart et al., 2006) for reasons which include dissociative amnesias (Şar et al., 2014a). The dual representation theory of PTSD (Brewin, 2001) assumes that many details of the traumatic experience are retained in the “situationally accessible memory” (SAM) rather than the “verbally accessible memory” (VAM). According to the multiple code theory of emotional processing, inhibition of a transition from the subsymbolic (patterns of sensory and visceral sensations and motoric activity associated with states of emotional arousal) to the symbolic (images and words) level of thinking (Bucci, 2007) which hinders the integration between emotion and cognition.

While Lacan's interest in trauma and the impact of “the real” was part of a meta-psychological model, Ferenczi (1932/1995) attempted to directly solve a clinical problem.This is shown in his Clinical Diaries (Kirschner, 2015). In his well-known paper which considered the interpersonal dynamics of the condition, (Ferenczi, 1932/1988) referred to the “confusion of tongues” between adult and child in the context of sexual abuse. Lacan's approach to trauma as the presence of the effect of unsymbolized real physical experiences seems to be in accordance with Ferenczi's notion of the unprocessed foreign body in the psyche (Garon, 1993 cited by Kirschner, 2015). Like Ferenczi's concept of foreign body (Kirschner, 2015), the Lacanian concept of an inassimilable “real” that cannot be contained in the symbolic (of speech) suggests an implantation of otherness in the psyche that produces effects.

According to the “Theory of Functional Dissociation of Self” (Şar and Öztürk, 2007), depressive phenomena, loss of interpersonal mutuality and functional somatic experiences are resistances of the “trauma-self” (Öztürk and Şar, 2016a). They require resolution in psychotherapy to repair the detaching of “sociological” and “psychological” selves. This is the point at which therapist and patient meet each other. In order to be therapeutic, such meeting should facilitate establishment of a “common language” not only between therapist and client but also between and across sociological and psychological selves (of both parties).

## Dissociation and attachment: personal and interpersonal

Dissociation is not only an intrapsychic and psychosomatic (embodied) but also an interpersonal phenomenon (Liotti, 2006). The latter contributes to the dynamism of dissociation in terms of the contextual factors affecting the condition of the individual. Hence both Liotti (2006) and Barach (1991) have underlined the role of interpersonal attachment disturbances in DID. Liotti (2006) proposes that pathological dissociation should be viewed as a “primarily intersubjective reality hindering the integrative processes of consciousness,” rather than as an intrapsychic defense against mental pain. Additionally, early defenses against attachment-related dissociation may lead to interpersonal controlling strategies that further inhibit the attachment system. Dissociative symptoms emerge as a consequence of the breakdown of these defensive strategies when exposed to events that activate the attachment system.

In the view of Liotti (2006), it was Bowlby (1973) who first hinted at the relationship between attachment processes and dissociation. Namely, Bowlby proposed that inadequate care-seeking interactions with primary caregivers could lead the infant to develop multiple internal representations of self and attachment figures (which he called Internal Working Models; IWM). One IWM becomes dominant in regulating interpersonal relationships in a certain context, while the other IWMs remain separated from mainstream conscious experience. The latter surface in stressful situations to regulate emotions and cognitions in a way that may be perceived as alien to the person's usual sense of self. In addition, sense of agency is influenced by this alteration.

Liotti (2006) suggests that the shifts among the multiple IWMs correspond to the drama triangle elaborated by Karpman (1968); i.e., the interactions between the main characters oscillate between the roles of the benevolent rescuer, the malevolent persecutor, and the helpless victim. The link between attachment theory and the drama triangle is represented in the model of attachment to the perpetrator (Ross, 1997) which allows the victim to achieve a subjective sense of control in the abusive condition. Hence, according to Liotti (2006), psychotherapy for pathological dissociation should be a phase-oriented process focused primarily on achieving attachment security, and should only secondarily deal with trauma. Therapeutic interventions aimed at disavoval of the “attachment to the perpetrator,” both inside the internal world and in external reality, are necessary to eliminate such destructive bonds. Disavowal of the attachment to the perpetrator, however, may require some type of trauma work.

Although the therapist would try to act as a secure base to improve alliance and symptom control (Cronin et al., 2014), this does not mean that the therapist's security would necessarily lead to the patient's security. Before treating trauma, the theapist cannot know what the client may perceive as sufficiently safe (Bromberg, 2006). Individuals who report high levels of dissociative experiences also present a negative view of themselves in attachment and close relationships (Şar et al., 2010a; Schimmenti, 2016). This may limit their potential to trust in themselves and to improve the quality of their relationship including the relationship with the therapist. Hence, if developmental trauma is not processed; i.e., a better level of integration and wholeness is not attained, attachment security may be difficult to achieve by highly dissociative individuals. This requires elimination of the resistances of the trauma-self (Öztürk and Şar, 2016a). The conception of the “sociological self” (Şar and Öztürk, 2007, 2013), however, serves to fit the needs of interpersonal attachment. The aspect of self desribed by this term is prone to fragmentation due to the conflictual demands of the external world. Hence, the trauma-self should be liberated from “guardianship” of the sociological self to operate in a problem-solving manner and on the basis of reality (Şar and Öztürk, 2005).

In his schema on three levels of object (interpersonal) relationships, Battegay (2008) mentioned “*fusion”* (a basic feeling of being “one” with the “object,” or as an alternative explanation, an intense feeling of attachment), “*active performances* (defense mechanisms) *of ego”* as two levels before a “*mature*” (reality-oriented) contact is possible. Projective identification serves as a regulator of interpersonal distance (e.g., by inducing anger as an aversive factor) in conditions where fusion becomes unbearable (Battegay, 2008). In fact, projective identification is based on interpersonal dissociation where the subject of emotions, thoughts, and behavior becomes contentious (Howell, 2003). The capacity of fusion may turn to a threat to individual autonomy in certain conditions, leading to loss of boundaries. We can observe this both in relation to crowds, communities, but also small groups (“group en masse,” Battegay, 2008) when they are under the influence of mass affect (which liberates capacities for fusion).

Individuals who become members of a cult or terrorist organization (e.g., the so called “Islamic State of Iraq and Syria”) and engage in destructive acts (e.g., “suicide bombing”) may be contemporary examples of malignant liberation of fusionary tendencies. Such identifications seem to be a consequence of extremely detached and expanded “sociological selves” which both enable and facilitate “mind control” (Vogt, 2012) conducted by any source of abusive power. “Sociological self” is also the origin of pathological and non-pathological possession experiences which constitute a currency of exchange with others in the transitional area between individual and society (Winkelman, 2011). They are perceived as external entities controlling the person. Unlike the individual alter personalities, they can affect and even “intrude” others as well; (hence, are entities “shared” in the community). Experiences of possession are associated not only with developmental traumatization but also with PTSD related to stressful events of adulthood (Şar et al., 2014b). Creating a cultural equivalent of the dissociative subtype of PTSD, they seem to be a way of coping with environmental threat.

## Internal and external phobias: the need—fear dilemma

The concept of trauma refers to a stressor located in the external world. However, this does not prevent the traumatic “experience” from having subjective components (Fischer and Riedesser, 1999). While prevention and recognition of trauma by the community are important, limiting the focus to the objective component of trauma would lead the therapist to helplessness in the face of the “cold facts.” Subjectivity, on the other hand, allows avenues of processing the traumatic experience via psychotherapeutic treatment. Thus, notwithstanding its focus on trauma due to environmental stress, this paper accords with the statement that “it is not possible to expiate the stressful external reality without solving the dilemma in the internal world of the patient” (Kalsched, 1996) because “the enemy who started on the outside is transformed into an inner torment” (Van der Kolk, 2011). The traumatized psyche becomes self-traumatizing (Kalsched, 1996, p. 5). Hence, the healing process has to be navigated from within.

In their “preliminary” paper, Breuer and Freud (1893/1999) stated: “We must presume rather that the psychical trauma -or more precisely the memory of the trauma—acts like a foreign body which long after its entry must continue to be regarded as an agent that is still at work; and we find the evidence for this in a highly remarkable phenomenon which at the same time lends an important practical interest to our findings.” While mentioning the relationship between internal world and external reality, Jung (1912) underlined dissociative aspect of this condition as well: “Neurosis is intimately bound up with the problem of our time and really represents an unsuccessful attempt on the part of the individual to solve the general problem in his own person. Neurosis is self division.”

DID and its subthreshold forms are characterized by “internal phobias” (Steele et al., 2001) against traumatic memories and alternate personality states which are complex derivatives of these memories, including anthropomorphisation, usually perceived as internal figures. This inner world constitutes an “internal family system” (Schwartz, 1995) comprised of members carrying roles and trauma-related “schemas” (Young et al., 2003). This is a “non-democratic” world characterized by oppression, rigidity, and dependency. Such relationships are also trasferred to the external world. Hence, frightening figures in the inner world of the patient co-exist with “internal self-helpers.” Members of the internal system are in relationship with each other as well as with significant others in the external world. However, internal phobias trigger distance to the content of the internal worldin the subject, rendering the dissociative amnesia noticable to the observer (e.g., intra-interview trance or amnesia) without facilitating entry of the interviewer into the fearful internal world of the patient (i.e., due to the phobia of the latter).

In purely descriptive screening studies, the most frequently seen personality disorders in DID are borderline, paranoid, compulsive, avoidant, and dependent types (Ellason et al., 1996; Kiziltan et al., 1998). This somewhat contradictory combination of traits which carry components of all three DSM-5 personality disorder clusters (i.e., A, B, and C) describes the traumatized person who lives in an alarmed “self-preservation” mode, rather than benefiting from a relaxed “self-regulation” mode (Ford, 2009). Compared to the non-dissociative depressive patients, patients with a dissociative disorder reported both preoccupied and fearful styles of attachment more frequently (Şar et al., 2010a). Representing D-type attachment (Liotti, 2006), this fits the constellation of the “need-fear dilemma” also known as Schopenhauer's “hedgehog's dilemma” (Schimmenti and Caretti, 2016); i.e., simultaneous need for and also fear of an event, object, or individual due to possible re-traumatization. This dilemma has been proposed to describe the immense need of the autistic schizophrenic individual for interpersonal contact but also the simultaneous anxiety and feeling of being threatened by close and intimate relationships (Burnham et al., 1969).

Dissociative individuals live under the influence of both negative self- and negative-other models which undermine any hope and will for emancipation (Şar et al., 2010a; Schimmenti, 2016). The “double bind” (Bateson et al., 1956) situation here represents a tendency to become entrapped in interpersonal relationships, which seems to be a consequence of loss of basic trust both in oneself and others due to the post-traumatic disillusionment (Fischer and Riedesser, 1999). Hence, threats against a sense of security and control may also originate from the internal world of the patients. Such experiences may constitute a chronic source of anxiety, and lead to diverse symptoms accompanying the strivings of the individual to ameliorate this fear.

Howell (2003) conceptualized pathological narcissism as a trauma-related condition and the interpersonal aspect of dissociation. The presence of overblown self-object representations in the internal world may be a consequence of non-availability of appropriate relationships in the external world. This corresponds to childhood neglect. Battegay (1987) objected also to conceptualization of pathological narcissism as a “personality disorder.” He proposed instead, classifying it as a narcissistic “neurosis” related to adversities of early childhood (neglect in particular). This problem is also valid in the opposite direction; i.e., attachment to internal self-objects prevents healthy interchange with the external world and interferes with true intimacy in close relationships. With the concept of “self-object,” psychoanalytic self psychology emphasized the interpersonal attachment and underlined the problem of interpersonal boundaries as well as disturbances of self-identity as a consequence of the “weak” self in pathological narcissism (Kohut, 1971).

Many patients with dissociative disorders unsuccesfully struggle alone for several years with their condition to achieve an improvement (self-reparation or self-treatment) before the appropriate diagnosis is made by a clinician (Öztürk and Şar, 2016b). It is a desperate struggle, because *the dissociative “ghetto”* (Frankel and O'Hearn, 1996) *of the internal world is self-perpetuating. It can not be solved without an intervention from the external world*; i.e., being “touched” (Kuechenhoff, 2007). This work is the essence of the treatment of the post-traumatic self. In his sensitive text on “clinical psychotherapy” for psychosis, Benedetti (1992) underlined the importance of the point at which a symbolic act of “transgression” on the part of the therapist became vital to achieving the treatment goal. A recent survey documented that experienced therapists reported that some type of boundary modification occurred in their treatment of post-traumatic conditions (Sachs, 2013). Careful assessment of the dynamics of such modifications requires the highest level of skill in psychotherapeutic practice.

## Being alone and with others: perceptual fluctuations

The interpersonal nature of dissociative disorders is misinterpreted by some clinicians (Chodoff, 1997) and researchers (somewhat strangely, in the name of an alleged “sociocognitive” model) as a proof for their iatrogenesis. In fact, dissociative disorders indeed have sociocognitive origins (as do several psychiatric disorders and psychological trauma itself). Hence a truly sociocognitive etiology neither excludes the role of psychological trauma in the origin of dissociative disorders, nor constitutes proof of iatrogenesis (Şar et al., 2013b).

The influence of the observer on the subject to be assessed is recognized even in exact sciences such as physics and computer programming. This cannot be expected to be different in clinical psychology and psychiatry, which work on subjectivities as well as objectivities. There are software bugs in computers which seem to disappear or alter behavior when one attempts to study it (Raymond, 1996); an effect called “Heisen bug” in remembrance of the thesis by Werner Heisenberg, the physicist who first asserted the observer effect of quantum mechanics, stating that the act of observing a system inevitably alters its state. One may also have different experiences about oneself when alone or in the presence of others.

Discrepancies in self-report and clinician assessment have been reported for BPD (Edell et al., 1990). In a study on college students who had either a dissociative disorder or BPD or both, there were significant differences between self-reported and clinician-rated symptom scores (Şar et al., 2014a, 2017a,b). Compared to dissociative disorders, BPD was related to an awareness of dissociative amnesia, depersonalization, and identity alteration in self-report more readily than the clinical interview. Participants with dissociative disorders were unable to report dissociative amnesia in self-assessment possibly due to “amnesia to amnesia” (Kluft, 1988). Hiddenness of the selected dissociative symptoms in BPD during clinical interview may be interpreted in two ways; i.e., a feature directly related to the psychopathology or the de facto diagnostic preference in the absence of self-report assessment. Nevertheless, underreporting of amnesia, identity alteration, and depersonalization-derealization in clinical interview may lead to overdiagnosis of BPD in patients with dissociative disorders (Şar et al., 2017a).

Such fluctuations in assessment may pertain to the self-system of the individual with a dissociative disorder (with or without BPD). This may involve hidden influences of non-executive personality states, or which may cause covert switching of distinct mental states during assessment contingent on perceptual alterations to traumatic memories (Beere, 2009a,b). Beere (2009b) has demonstrated that as the dissociative psychopathology entered the DID range (the most severe end of the dissociation spectrum), reports of amnesia appeared to decrease (although it is known clinically that there is more amnesia among those manifesting DID than manifested in this particular subgroup). The presence of an interviewer clearly represents a situation that is the model setting of interpersonal attachment, which has a crucial importance for the studied population (Liotti, 2006). Apparently, the presence of a clinician (interpersonal situation) or aloneness during assessment may have distinct effects on the mental status of the evaluated subject. If so, what is the relationship between this effect and the two diagnostic patterns?

Individuals with BPD were reported to have significantly better performances in the Reading the Mind in the Eyes Test (RMET). This is a measure of capacity to discriminate the mental state of others from expressions in the eye region of the face (Fertuck et al., 2009). Antidepressant medication status, PTSD co-occurrence, current vs. past depression, and childhood physical or sexual abuse did not predict this observation. Indeed, BPD seems to be related to a greater “interpersonal (external) phobia” and to increased vigilance against perceived threats in interpersonal situations. This would lead to a preponderance of memory continuity in the self-system during a clinical interview, e.g., by taking over of executive personality states. Interestingly, based on data collected from clinical and non-clinical subjects, Kaehler and Freyd (2009) found that higher “betrayal” traumas are associated with greater BPD characteristics. Among college students, none of the childhood trauma scores except SA was correlated with clinician-rated dissociative amnesia; however, such correlation existed for all trauma types in self-report (Şar et al., 2014a). Nevertheless, all types of childhood trauma and both types of dissociative amnesia were correlated with total number of criteria of BPD as the common final pathway representing the severity of the condition, and, even that of dissociation (Şar et al., 2017b).

Underlining the significance of “betrayal” (Freyd, 1994), in a neurobiological study on adolescent girls with PTSD due to severe sexual abuse (Mutluer et al., in press), those abused by their biological father or brother (i.e., perpetrator in “closer” relationship with the victim) reported more dissociative amnesia and absorption compared to victims of other perpetrators. They also had smaller left anterior cingulate while right anterior cingulate was larger in coitus group (i.e., more severe abusive act) compared to those with other types of SA. Both these psychological and neurobiological phenomena may point to a possible strivings of the organism to manage the psychological pain (e.g., by denial, attachment to the perpetrator, dissociative amnesia, and estrangement).

## Reality testing and distortion of reality: dissociation and loss of insight

Dissociation is a way of dealing with reality (Şar and Öztürk, 2005) while trying “to link the overwhelming with the unbearable” (Schimmenti and Caretti, 2016). Psychological trauma represents a breaking point in human life characterized by a drastic change in reality which requires the re-adaptation of the affected individual to the emerging condition. Psychological treatment of such conditions should assist the subject to transition “between realities.” Such transition may entail creation of a new self-definition for an upcoming stage of life while integrating the past, where “lost” identities are sometimes also “found” during the process of psychotherapy. Interpersonal developmental trauma occurs in context of distortion of reality by the perpetrator; i.e., betrayal. Betrayal trauma theory suggests that dissociation is an adaptive response to childhood abuse, which allows survival by enabling the child to maintain attachment to a figure vital to her/his development (Freyd, 1994).

According to pioneering trauma clinician and researcher Janet, mental health is characterized by a high capacity for integration which unites a broad range of psychological phenomena within one personality (Van der Kolk and Van der Hart, 1989). To achieve this, and in addition to a general capacity for synthesis in the central nervous system, “realization” (not only accepting and recognizing experiences as real but also contextualizing them in order to obtain meaning) of experiences is required. This requires “personalization” (claiming ownership) and “presentification” (ability to differentiate between past, present, and future) of the experience. Traumatic stress jeopardizes these abilities, thereby leading to dissociation with diminished awareness, comprehension, and self-control. In both normal and abnormal functioning, reflexive self-awareness is crucial but can be disturbed by the tension that arises from coordinating subjective and objective perspectives about oneself.

In his psychoanalitic essay “Standing in the Spaces,” Bromberg (1998) elaborated the “normal multiplicity” of the self. Paradoxically, he considers dissociation primarily as the means through which the individual maintains personal continuity, coherence, and integrity of the sense of self in front of the unbearable reality. Dissociation allows the existence of several different (subjective) versions of reality within one person. Thus Kluft (1993) once called DID “multiple reality disorder” (rather than multiple personality disorder) and referred to “alternating reality states” (Kluft, 2003). Somewhat similarly, Chefetz (2004) refers to identity alteration in DID as “isolated subjectivities.” Paradoxically, distinct or “alter” personality states are not disintegrated structures only but they also represent a striving of re-establishment of the lost unity (Şar and Öztürk, 2005).

Being able to differentiate fantasy from reality is critical in achieving the integrative mode of consciousness (Öztürk and Şar, 2016b). “Trance-logic” (i.e., the tolerance and/or rationalization of logical inconsistency while in a hypnotic state) which is a core aspect of the cognition of DID patients (Loewenstein, 1993), allows the patient to adjust to “normal” daily life while maintaining beliefs which are not only inconsistent with external reality but may be contradictory among themselves.This is a phenomenon resembling one of Bleuler's (1911) observations of schizophrenia (which does not reference dissociation)—namely, “double bookkeeping” (doppelte Buchführung). Hence, the dissociative patient's claim to have another person inside him/herself, or to have more than one personality, should not be considered a delusion *per-se*.

An intriguing aspect of such trance-logic that has important behavioral consequences is related to variations in the belief that dissociative identities are separate. At the extreme end, one finds patients who view dissociative identities as almost like “real-world friends.” In some cases, the “murderous” intention of a “persecutory” part against the “host” personality may lead to and be equated with a suicide attempt. This may even lead to a completed suicide due to failure to comprehend that both parts belong to the same person. Putnam (1989) has called this phenomenon “internal homicide.” Such experiences can also mimic paranoid conditions, as when a persecutory dissociative identity is projected onto a person or directly perceived as a hallucination in the outside world. From an external vantage point, this would look like a delusion of persecution unless the dissociative identities involved are identified as the source of the experience. Nevertheless, with the exception of this “circumscribed psychosis,” even these patients do not lose reality testing in other domains of life.

In an empirical study, patients with a dissociative disorder had better cognitive insight than patients with a schizophrenic disorder, and did not differ from those with obsessive compulsive disorder or major depression (Şar et al., 2012). However, unlike the latter two groups, dissociative patients were similar to the schizophrenic group in terms of heightened “self-certainty.” Nevertheless, the “self-reflection” scores of patients with dissociative disorder were better than those of the schizophrenia patients (which placed their overall cognitive insight in the non-psychotic range). This “dissociation paradox” (Şar et al., 2012) is possibly attributable to defensive persistence by the traumatized individual who perceives threats in their environment. Interestingly, a recent study on a college population, participants with BPD tended to report “detachment from reality” more readily than those with a dissociative disorder (Şar et al., 2017b).

The American Psychiatric Glossary defines reality testing as the ability to evaluate the external world objectively and to differentiate adequately between it and the internal world (Stone, 1988). Impaired reality testing is one of the major hallmarks of psychosis. Although the “internal world” is especially complex in DID, it is rarely confused with “external reality.” Where this happens, it is only in a time-limited and/or circumscribed fashion.

Reality testing is intact in patients with dissociative disorders except during dissociative psychotic episodes (where the latter may constitute crisis states superimposed on the ongoing dissociative pathology) or those conditions when the patient's belief on the separateness of personality states takes a delusional scope. Loss of insight due to chronic or acute dissociation can also have forensic implications in criminal cases (McFarlane, 1998).

## Crisis of security and self-control: turning “inside out”

Concerns about security and control may lead to a crisis for dissociative patients which may interrupt their daily functioning including transient impairment of reality testing. Two syndromes may dominate the frontline clinical picture of a patient with DID in this regard: functional neurological (conversion) symptoms and acute dissociative reactions. The two conditions represent the most dramatic versions of sensori-motor and cognitive-emotional dissociation. Both may co-occur and, even, may be superimposed on DID (Nijenhuis et al., 1998). They are frightening for the affected patients as well as for their close social network. For example, with their seemingly life-threatening nature, sensori-motor dissociative symptoms such as non-epileptic seizures constitute a medical emergency (Şar et al., 2007). In a clinical series of patients with dissociative disorders, somatic symptoms predicted suicidal ideation (Öztürk and Şar, 2008). Many patients with DID are admitted to psychiatric services subsequent to treatment-resistant and dramatic conversion symptoms including non-epileptic seizures (Tezcan et al., 2003; Şar et al., 2004).

Severe acute dissociative reactions with psychotic features have been known as hysterical or as reactive dissociative psychosis (Van der Hart et al., 1993) which may resemble a delirium, mania, schizophrenic disorder (Şar and Öztürk, 2009). Dissociative psychosis of patients with DID consists of “revolving door crisis” (Putnam, 1989) whereby the alter personalities compete for control (Tutkun et al., 1996) or a temporary breakdown of dissociative barriers leading to a “co-consciousness crisis” (Kluft, personal communication, 1995).

Nevertheless, acute sensori-motor or cognitive-emotional crises serve as a “cry for help” in difficult contexts including cessation of threats of an abusive environment. Thus, they constitute an “exit” strategy of the “system” (sometimes the last before a collapse which leads to attempted or completed suicide). Such crises may also occur in context of power struggle or a threat to attachment. Significantly, such struggle may occur in the “internal world” of the patients (usually between personality states in conflict) as well as in the external world (usually with family members and/or further significant others). Nevertheless, both “worlds” may trigger each other. Regaining a sense of security and control requires at least a temporary balance between them usually obtained by interventions in both spheres.

## Cultural preponderance of universal patterns: extratensitivity and introtensitivity

Interaction between internal and external worlds occurs in the context of the cultural environment. In a single case study on DID using the Rorschach projective test in Turkey, the “extratensitivity” of the patient diminished after psychotherapeutic treatment leading to integration (Şar et al., 2002). The concurrent diagnosis of BPD dropped as well. These findings contradict the reports on North American dissociative patients who appear to have a predominantly internally focused coping style (Armstrong and Loewenstein, 1990). Nevertheles, a study on identity alteration in Turkish college students yielded separate dimensions representing either “mental intrusions from within” or those characterized by “externalization” of the dissociative experience (Şar et al., 2017a).

“Introtensive” means emphasizing thought, fantasy, and imagination, or inner life (internally focused coping style). A subject who focuses upon affective response to the environment as his base of experience is called “extratensive” (externally focused coping style). The change in Rorschach responses of this patient is in accordance with impaired impulsiveness after treatment. Interestingly, in a comparison of Turkish and Dutch patients with DID, large differences existed between the two groups in BPD diagnostic criteria met rather than the core dissociative symptoms (Şar et al., 1996). Namely, Turkish patients reported intense anger and lack of control of this emotion, chronic feelings of emptiness and boredom, efforts to avoid abandonment, and intense but unstable relationships more frequently than Dutch patients. In turn, Dutch patients reported frequent mood swings, physically self-damaging acts, identity confusion, and impulsive and unpredictable behavior more frequently than Turkish patients. Some type of affect dysregulation was common to both groups (Chefetz, 2005).

The dichotomy of internal and external phobias seems to be operative here. Apparently, BPD criteria represented both the personal and interpersonal aspects of dissociation among these patients (Şar, 2006). Being part of a relational culture, Turkish patients had more symptoms in the interpersonal area inspiring attachment disturbances (behavior influenced by “external interpersonal phobias”) more frequently than Dutch patients who seemed to struggle in their internal world individually (behavior infuenced by “internal phobias”). These differences in predominant post-traumatic response types may be related not only to cultural factors affecting symptom presentation (e.g., overall lifestyle, drug abuse, family relationships) and sanctions on disclosure of trauma, but also to etiological factors.

Awareness about the traumatic nature of life experiences may be affected by the cultural environment. This is apparent in a study of women with somatization disorder living in a semi-rural region of of eastern Turkey (Taycan et al., 2014). Participants in this study had been exposed to every type of trauma endemically but had limited awareness of the nature of their environment. As well as functional somatic complaints, possession (i.e., non-delusional passive influence experience attributed to external entities) rather thanclassical cognitive-emotional dissociation was among main predictors of the diagnosis.

Trauma may be “invisible” as observed in overprotective childrearing as a type of interpersonal intrusion. This is known to be relatively common in Turkey, including among the well-educated middle and middle-upper class. For example, adolescents with DID and its subthreshold forms did not differ significantly from non-dissociative adolescent psychiatric outpatients on childhood abuse and neglect and family dysfunctionality scores as assessed by self-report measures (Şar et al., 2014c). However, the dissociative group had more comorbid separation anxiety disorder compared to controls possibly due to attachment disturbances. The instrument used to assess childhood trauma did not cover overprotection—overcontrol by parents. In fact, this self-compensatory behavior of traumatized parents leads to intergenerational transition of trauma. This style not only threatens interpersonal boundaries and private individual spheres, but is overwhelming for the rising generation due to implied control by feelings of guilt (Kogan, 2007). Hence, these families may be considered as “apparently normal” (Öztürk and Şar, 2005) which, in fact, comprise of members with traumatic antecedents, affect dysregulation, and disturbed communications.

Both the experience and expression of traumatic stress may be influenced by sex and gender. The latter is heavily shaped by culture. A recent study documented examples of these differences as observed in variability of reaction types of traumatic stress among male and female Yazidi refugees with PTSD in Turkey (Tekin et al., 2016). Both genders had been exposed to war-related trauma. Women reported flashbacks, hypervigilance, and intense psychological distress due to reminders of trauma (undermodulation of emotions) more frequently than men. On the other hand, men reported feelings of detachment or estrangement from others (overmodulation of emotions) more frequently than women. More depressive women than men reported feelings of guilt or worthlessness. It is not yet known whether such differences between women and men are based on gender (socio-culturally formed roles) or sex (biologically formed differences).

The shape and functions of crisis episodes superimposed on a trauma-process constitute a further culture-sensitive sphere. Both the prevalence of non-epileptic seizures as well as acute dissociative reactions with psychotic symptoms seem to differ widely in different parts of the world. This points to the strong cultural aspects of these conditions (Şar et al., 2009; Martinez-Taboas et al., 2010). To a degree unusual for other psychiatric constructs, cultural sensitivity in experience, and/or assessment of dissociative disorders (Şar, 2006; Lewis-Fernandez et al., 2007) is represented even in differences between the official diagnostic manuals of psychiatric disorders such as the DSM-5 (American Psychiatric Association, 2013) and the ICD-10 (World Health Organization, 1992). While the DSM-5 is more sensitive in describing chronic cognitive-emotional dissociation, the ICD-10 is focused on acute and sensori-motor dissociation. Both systems ignore the acute dissociative reaction with psychotic features. Such oversight impedes better understanding of the relationship between crisis conditions and chronic underlying psychopathological processes. At the same time, this relationship may illuminate the diverse ways of handling conflicts in the internal and external world as represented in various cultures. Social origin of such conditions may exceed that of the individual and family during a historical period of drastic social change (Krüger et al., 2007; Hegeman, 2013; Demirbugan, 2016).

## Becoming “one-self”: overcoming the fear of loneliness

Dissociation is not a static but a dynamic condition. Individuals suffering from posttraumatic dissociation do not experience only discontinuities and/or disruption of their mental functions. Rather, they experience simultaneously an intense striving to achieve the normal integration of these to attain the sense of experience of wholeness (Wilson, 2006; Tagliavini, 2014). Associations tend to be established between disrupted structures of the mind which may remain hidden in the eyes of the observer in the first instance. Integration requires connection, inside and outside. Paradoxically, distinct or “alter” personality states in DID do not solely comprise disintegrated structures. They also represent a striving for re-establishment of the lost unity (Şar and Öztürk, 2005). In fact, psychotraumatology is a discipline requiring dialectical thinking (Fischer and Riedesser, 1999); i.e., opposites may coexist (Şar, 2015a). To this extent, “alter” personalities in DID also constitute an attempt to bridge the gap between the detached “sociological” and “psychological” selves (Öztürk and Şar, 2016b).

Traumatic stress is in a relationship with formation as well as transformations and disturbances of self-identity (Wilson, 2006; Putnam, 2016; Şar, in press). Firstly, trauma may affect memory which influences perception of one's life (Brewin, 2011). Mental intrusions (e.g., vivid memories) and omissions (e.g., amnesias) may undermine one's sense of self and agency. What makes the influence of trauma on self-identity more complex is that the *naturally wired protective response* of the human organism to chronic developmental stress; i.e., the division of mind to save the unique aspects of the individual. Thus, in effect, dissociation serves to “safeguard” the potential of regaining the hidden preserved treasure. The cost of this dividedness is self-estrangement. Hence, re-claiming one's authentic self-identity remains the basic mission of psychotherapy for trauma-generated conditions.

Given the dual nature of the interplays in, as well as between, the internal world and external reality, mental integration as both the means and goal of healing needs to lessen internal and external fears and re-establish mutuality (Erikson, 1950/1963) between the internal “world” (psychological “reality”) and external (sociological) “reality.” This is also while retaining the boundaries between them (Fonagy et al., 2002). The “Theory of Functional Dissociation of Self” (Şar and Öztürk, 2007, 2013) explores and proposes avenues of integration for dividedness of human consciousness which has outlived its purpose of *ad hoc* adjustment to external reality.

## Author contributions

The author confirms being the sole contributor of this work and approved it for publication.

### Conflict of interest statement

The author declares that the research was conducted in the absence of any commercial or financial relationships that could be construed as a potential conflict of interest.
